# Microglial Maturation and Functional Heterogeneity: Mechanistic Links to Neurodevelopmental Disorders

**DOI:** 10.3390/ijms27031185

**Published:** 2026-01-24

**Authors:** Pariya Khodabakhsh, Olga Garaschuk

**Affiliations:** Institute of Physiology, Department of Neurophysiology, Eberhard Karls University of Tübingen, 72074 Tübingen, Germany

**Keywords:** microglia, neurodevelopment, synaptic pruning, circuit maturation, epileptogenesis, autism spectrum disorders

## Abstract

As the brain’s resident macrophages, microglia on the one side are increasingly recognized as essential players in discrete developmental stages, where immune, metabolic, and activity-derived signals are coordinately integrated to guide brain development. On the other side, the precise temporal and molecular coordination of microglial maturation is imperative for the structural and functional integrity of the developing central nervous system (CNS). In this review, we synthesize recent data that reposition microglia from a uniform population of immune sentinels to temporally programmed and regionally specialized regulators of circuit maturation. This involves dissecting the embryonic origins and migratory pathways of microglial progenitors in mouse and human systems and illustrating how gradual transcriptional and morphological maturation aligns the biology of microglia with progressive phases of neurogenesis, synaptic fine-tuning, myelination, and vascular stabilization. In addition, we discuss how individual gene mutations, inflammatory insults during perinatal life, and environmental disturbances intersect with these temporal programs to alter microglial phenotypes and compromise circuit formation. With a special emphasis on epilepsy and autism spectrum disorder, often sharing the common etiology, we illustrate how early malfunction of microglia may drive neural network dysfunction.

## 1. Introduction

The complex architecture of the CNS is defined by diverse cell populations. Herein, microglia are distinguished as unique resident myeloid cells, as well as CNS parenchyma-specific macrophages [1]. Although individual microglia are often long-lived, as a population they are dynamically regulated by balanced cycles of proliferation and apoptosis. This guarantees both demand-driven self-renewal and population stability throughout life [2,3]. These cells display remarkable morphological plasticity, shifting from immature ameboid microglia that enter the brain during embryonic development, to a highly ramified, process-rich form under homeostatic conditions in adulthood, and reverting to a motile, ameboid morphology in response to insults such as infection or trauma. It is now acknowledged that this dynamic response is not simply a binary switch of “activation”. Instead, microglia can enter a wide range of context-specific states, each marked by distinct transcriptional signatures, morphology, and specialized functional outputs [1,4].

Besides functioning as immune sentinels, thus mediating inflammation, fighting off invasive pathogens, clearing cellular debris, and regulating tissue repair, microglia are indispensable for proper brain homeostasis. By promoting neurite extension and synaptogenesis, regulating synaptic pruning to enhance connectivity, and supporting neuronal survival through trophic factors, microglia play a crucial role in neuro-glial development and adult brain homeostasis [1,5]. According to recent single-cell transcriptomic studies, fetal and adult microglia exhibit distinct transcriptional and functional characteristics. Consistent with their roles in supporting neuronal development and circuit formation, fetal microglia express higher levels of genes associated with neurogenesis, axon guidance, and synapse formation [6,7,8], whereas adult microglia express many genes related to immune surveillance, homeostasis, and inflammation, consistent with their primary functions in maintaining CNS integrity and responding to injury or disease [9,10,11]. This observation is well supported by both human and mouse studies [8,12].

In this review, we examine the developmental and functional diversity of microglia in rodents and humans, along with their spatial dynamics and signaling mechanisms, tracing their progression from embryonic origins to region-specific specialization. Combining insights from developmental biology and neuroimmunology, we explore how the temporal and molecular coordination of microglial function shapes the developing brain, and how the perturbations in these processes may lead to epilepsy and autism spectrum disorder.

## 2. The Emergence of Microglia: Origins and Lineage Specification

Developmental origin of microglia is distinct from that of other glial cells, such as astrocytes and oligodendrocytes, which arise from the neuroectoderm. By contrast, microglia are derived from mesodermal erythromyeloid progenitors (EMPs) that emerge in the yolk sac during primitive hematopoiesis, around embryonic day (E) 7.25–7.5 (Table 1). These progenitors express CSF1 receptor protein (CSF1R), PU.1 (SPI1), and RUNX1, but notably lack the hematopoietic stem cell regulator c-MYB (c-MYB^−^ cells), which sets them apart from later myeloid lineages [13,14]. Through lineage-tracing studies, these early yolk sac-derived macrophages have been shown to migrate into the developing neuroepithelium before the establishment of the blood-brain barrier (BBB), where they differentiate into brain-resident microglia. The transcription factors RUNX1, PU.1, and IRF8 are essential for their early specification and survival [15,16].

Recent single-cell transcriptomic profiling of human embryonic macrophage progenitors has identified MRC1^+^ CD163^+^ populations within the yolk sac at Carnegie stage (CS) 11, displaying transcriptomic signatures nearly identical to microglial progenitors found in the embryonic mouse brain at the same stage [17], thus supporting a conserved yolk sac-derived origin across species. A secondary population of CD34^+^ MYB^+^ macrophages appears later (around CS 17) in humans, corresponding to the late mouse EMPs that seed the fetal liver and give rise to monocytes, further reinforcing parallels between human and murine hematopoietic waves [17,18].

**Table 1:** Developmental origin and CNS integration of microglia in mice and humans.

Developmental Stage	Timing (Mouse vs. Human)	Key Molecular Markers/Regulators	Morphology & Functional Significance	Key References
Primitive yolk sac EMP wave	Mouse: E7.25–E7.5 Human: postconceptional weeks (PCW) 2–3	CSF1R, PU.1 (SPI1), RUNX1, c-MYB^−^	Ameboid macrophage progenitors. Microglial lineage, distinct from neuroectodermal glia and HSC (hematopoietic stem cell)-derived myeloid cells. Foundation for lifelong CNS residency.	[13,14]
Hoxb8^+^ transient definitive EMP wave	Mouse: E8.5–E10.5 Human: PCW 4–5 (inferred)	c-MYB, Hoxb8, Runx, CSF1R	Transient microglia-like population. Impacts cortico-striatal circuit maturation. Loss leads to compulsive and anxiety-like Behaviors.	[19,20]
Definitive HSC-derived myeloid progenitors	Mouse: ≥E9.5 Human: PCW 5–7	c-MYB, Hoxb8, Runx, CSF1R, CD206 (Mrc1)	Primary source of CAMs. Minor contribution to parenchymal microglia. Plastic, allow partial lineage overlap.	[18,21,22]
Migration	Mouse: E9.5–E14.5 Human: PCW 4–10	CXCR4-CXCL12, CX_3_CR1-CX3CL1, integrins	Highly motile ameboid cells. Chemokine- and ECM-guided migration. Rostral-caudal colonization of neurogenic regions.	[23,24,25]
Entry into CNS	Mouse: ~E9.5 Human: PCW 4–12	CSF1-CSF1R, CX_3_CR1	Dual entry routes: trans-tissue (meninges, ventricles) and trans-vascular. Occurs before BBB maturation. Depends on the functional embryonic vasculature.	[13,14,26]
Establishment as native CNS cell (embryonic)	Mouse: E12.5–E18 Human: PCW 8–20	RUNX1, PU.1, IRF8, early TREM2	Ameboid-to-ramified transition. Integration into parenchyma. Regulation of apoptosis, neurogenesis, and early synaptic surveillance.	[15,27]
Establishment as native CNS cell (postnatal/adult)	Mouse: P0–P21 and adulthood Human: infancy to adulthood	Sall1, Tmem119, P2ry12, Hexb, Siglech	Fully ramified, territorially stable microglia. Activity-dependent synaptic pruning. Long-term immune surveillance and Homeostasis.	[28,29,30]

Beyond the initial EMP-derived lineage, a “transient definitive” wave of myelopoiesis arises around E8.5, generating c-Myb-dependent Hoxb8^+^ Runx^+^ Csf1r^+^ immature macrophages (sometimes termed A1 cells) (Table 1). These cells migrate through the aorta-gonad-mesonephros (AGM) region and fetal liver before entering the developing CNS [20]. Although their contribution to the adult microglial pool is minor, their functional importance is notable: mice lacking Hoxb8-derived microglia display compulsive grooming and anxiety-like behaviors due to disrupted cortico-striatal circuit maturation. Hoxb8 expression peaks embryonically and diminishes postnatally, suggesting that this lineage acts primarily during periods of synaptic refinement and circuit development [19,20].

A third wave of hematopoiesis occurs around E9.5, when c-Myb^+^ Hoxb8^+^ Runx^+^ Csf1r^+^ HSCs (A2 cells) emerge in the AGM and expand within the fetal liver (Table 1). These progenitors give rise to long-lived tissue-resident macrophages, including subsets that populate the CNS [18]. Intriguingly, A2 progenitors can be subdivided into CD206 (Mrc1)-expressing and non-CD206-expressing populations, with CD206 being a canonical marker of CNS-associated macrophages (CAMs). Fate-mapping studies reveal that CD206^+^ progenitors can generate both CAMs and parenchymal microglia [21,22]. Further supporting this view, a subset of intraventricular CAMs has been shown to invade the pallium around E12.5, revealing at least two parallel seeding routes into the developing brain [31].

At the molecular level, microglia and CAMs exhibit distinct gene expression profiles reflecting their specialized roles and microenvironments. Microglia are characterized by high expression of *Tmem119*, *P2ry12*, *Sall1*, *Hexb*, *Siglech*, *Slc2a5*, *Fcrl2*, and *Trem2* genes (Table 1) associated with neuronal communication, immune homeostasis, and lipid metabolism [27,28,29,30,32]. Conversely, CAMs exhibit elevated levels of *Cd206*, *Lyve1*, *Cd163*, *Siglec1*, and *Ms4a7*, which are associated with vascular interactions, antigen clearance, and inflammatory signaling [29,33]. Despite these transcriptional and spatial differences, recent single-cell and fate-mapping analyses suggest that the developmental boundaries between microglia and CAMs are more fluid than previously believed. Both populations may arise from overlapping progenitor pools and exhibit limited interconversion potential during embryogenesis or under pathological conditions [22,34,35].

In humans, microglia follow a broadly comparable trajectory (Table 1). Yolk sac-derived progenitors emerge around PCW 2–3 and invade the forebrain by PCW 3–4, preceding the onset of large-scale neurogenesis [36,37].

## 3. Becoming a Native of the CNS

### 3.1. Migration Routes and Entry to the Developing CNS

Microglia represent one of the first cellular lineages to colonize the CNS, a process that is completed before the maturation of the BBB [14,26]. Despite the rapid proliferation of macrophage progenitors within the extraembryonic yolk sac, no myeloid cells are detected in the murine CNS before approximately E9.5 (Table 1). This infiltration coincides precisely with the initiation of embryonic systemic circulation, indicating a prerequisite role of functional vasculature for the migration and entry of yolk sac-derived progenitors into the developing brain [14,26]. Using in vivo intravital microscopy in mice, it has been confirmed that between E9.5 and E14.5, Csf1r^+^ yolk sac-derived macrophage progenitors migrate intravascularly into the nascent brain and subsequently colonize it in a rostral-to-caudal gradient [23]. The dependency of microglial colonization on a functional embryonic vasculature has also been underscored by genetic and experimental models in which circulatory impairment significantly diminishes CNS infiltration by yolk sac-derived macrophages [13,14].

Microglial progenitors invade the CNS via two principal entry routes: trans-tissue and trans-vascular. Trans-tissue invasion occurs through the leptomeningeal and ventricular surfaces, whereas trans-vascular infiltration involves direct migration through the developing vascular lumen [13,14,34]. These processes are thought to be regulated by trophic signaling through the CSF1-CSF1R axis (with CSF1R being highly expressed by microglia as well as perivascular, meningeal and choroid plexus macrophages) (Table 1), which governs progenitor attraction and survival [38]. The spatial distribution and precise positioning of microglia within the developing CNS are orchestrated by a complex interplay of chemokine signaling, extracellular matrix interactions, and cell-cell communication that together guide their migration into neurogenic niches and emerging cortical structures.

The CXCL12–microglial CXCR4 axis establishes chemotactic gradients that guide progenitors toward CXCL12-rich ventricular and subventricular zones, enabling bidirectional migration between the meninges and cerebral wall and promoting colonization of neurogenic niches [24]. Fractalkine-CX_3_CR1 signaling, driven by neuronal CX_3_CL1, further refines regional patterning by directing CX_3_CR1-expressing microglia into specific neural domains; loss of CX_3_CR1 delays their recruitment and spatial organization [39]. In parallel, integrin-mediated adhesion to extracellular matrix components such as fibronectin supports talin-1-dependent transmigration along the pial surface, complementing chemokine-guided movement [25]. Finally, cues from the neuroepithelium fine-tune local positioning, apoptotic cells release macrophage migration inhibitory factor (MIF) to stimulate recruitment and proliferation, while neural progenitors secrete attractants that draw microglia into active neurogenic regions [25,40]. Although the general migratory framework is well described, the molecular cues defining the timing, lineage specification, and selective colonization of microglial niches remain incompletely understood [34].

In humans, microglial colonization follows a similar developmental trajectory, though it occurs over a more protracted timescale. Microglial colonization of the human brain begins with the appearance of the cells in the forebrain around PCW 4, following the emergence of yolk sac-derived progenitors. This initial phase is supported by immunohistochemical evidence showing ameboid microglia entering the telencephalon and diencephalon via the meninges, choroid plexus, and ventricular zone, rather than through the vasculature [41,42]. By PCW 5, microglial precursors are evident around mesenchymal capillaries encasing the developing brain, suggesting a vascular route like that observed in mice [3]. However, the first confirmed evidence of trans-vascular entry into the human parenchyma occurs only around PCW 10–12, implying that earlier colonization phases may rely on alternative, nonvascular pathways, potentially involving the meninges or choroid plexus, as well as possible entry from the ventricular lumen [36,41,42].

### 3.2. Regulatory Mechanisms Governing Microglial Population Dynamics and Density

Microglial proliferation during CNS development is governed by a tightly coordinated interplay of trophic signaling, transcriptional regulation, and spatial constraints. Following their initial entry into the embryonic mouse brain around E9.5, microglial progenitors establish a sparse, developmentally regulated distribution across the cerebral wall that closely follows cortical maturation [14,23]. In the developing mouse cortex, the number and density of microglia increase with embryonic age, reflecting a true colonization process that is not solely attributable to cortical area expansion. Quantitative analyses demonstrate that microglial density rises substantially, by approximately sixfold, from E10.5 to E17.5, with statistically significant increases at specific intervals (notably from E10.5 to E11.5 and again after E14.5) [23]. After E14.5, there is a rapid increase in microglial cell number and density, but the proportion of proliferating microglia declines sharply, suggesting that this second phase is dominated by additional microglia entering the parenchyma from peripheral sources [23,31]. This biphasic pattern is consistent with observations in other regions of the embryonic mouse CNS, such as the retina and spinal cord [23].

As the BBB matures and achieves functional integrity by the end of the second postnatal week, the entry of microglial progenitors into the CNS from the periphery ceases. Thus, microglial cell density peaks around postnatal day 14 (P14), then briefly declines between postnatal weeks 3 and 6. This temporary decrease indicates a crucial refinement phase where excess microglia are eliminated through apoptosis, and proliferative activity decreases [43,44,45]. This process is tightly synchronized with key neurodevelopmental milestones, including synaptic pruning and circuit remodeling [3,46,47]. Notably, this decrease in density of microglia persists even in the presence of elevated trophic support (i.e., CSF-1), indicating that intrinsic maturation programs, rather than extrinsic growth factor availability, govern this phase of microglial refinement [46]. Thereafter, the population stabilizes and is sustained throughout life by slow, homeostatic self-renewal of resident mature cells, reflecting the remarkably long (months-to-years) lifespan of microglia both in mice and humans [2,48].

Microglial CSF1R receptors are key for their proliferation and survival from the earliest stages of embryonic colonization through postnatal maturation and into adulthood, with CSF1R ligands CSF1 and IL-34 providing complementary, region- and stage-specific support. CSF1 is critical for microglial colonization during embryogenesis, while both CSF1 and IL-34 are required for microglial maintenance beginning in early postnatal development. Their non-redundant, regionally distinct roles are evident in that neither ligand alone can fully compensate for the absence of the other, and only CSF1R ablation eliminates all microglia [38,49,50,51]. Transcriptional regulation of Csf1r is further refined by the Fms intronic regulatory element (FIRE) enhancer, whose deletion disrupts yolk sac progenitor expansion and microglial maturation [52].

In humans, multiple lines of immunohistochemical, pathological and genetic evidence confirm that biallelic loss-of-function CSF1R mutations result in near-complete absence of microglia in the CNS, and are associated with severe developmental brain anomalies, including corpus callosum agenesis, white matter abnormalities, and calcifications [53,54]. Such patients present early in life with global developmental delay, therapy-resistant epilepsy, and progressive neurological decline. The phenotype is distinct from the adult-onset leukoencephalopathy seen in heterozygous CSF1R mutations (adult leukoencephalopathy with axonal spheroids and pigmented glia), and includes additional features such as skeletal dysplasia, consistent with the role of CSF1R in both microglial and osteoclast development [54,55].

Transforming growth factor-β (TGFβ) signaling further contributes to embryonic, but not postnatal, proliferation, as deletion of Tgfbr2 during mid-gestation markedly reduces Ki67^+^ microglia, indicating a developmental time window of dependence [56,57]. TREM2 and its adaptor DAP12 (TYROBP) promote microglial proliferation and survival by activating the AKT-mTOR and β-catenin pathways, particularly in lipid-rich or immune-activated environments [58], whereas Fc receptor (FcR)-mediated activation via Bruton’s tyrosine kinase provides an additional, though context-specific, proliferative input [59].

Beyond molecular cues, the spatial microenvironment plays an equally crucial role in regulating proliferation. Microglial density is closely coupled to brain growth, with cells responding to mechanical tension and space availability via contact inhibition mechanisms that maintain optimal intercellular distances (approximately 40–50 μm). As microglia clonally expand, they compete for available space, and once local density reaches a threshold, contact inhibition limits further proliferation. This drives the transition from clustered to mosaic distribution and ensures even coverage of the parenchyma [37,60]. Unoccupied niches rich in trophic factors, such as CSF1 and IL-34, act to promote the local expansion until spatial equilibrium is reached, and therefore support the formation of a uniform, mosaic-like microglial network [60]. These biomechanical influences integrate with the transcriptional programs, coordinated by factors that include E2F, Klf/Sp, Nfy, and Ets, which regulate cell-cycle gene expression and modulate proliferative capacity according to regional cues and tissue growth dynamics [11,61]. Collectively, these molecular, transcriptional, and biomechanical pathways define a self-regulating framework that balances expansion with spatial organization throughout brain development.

### 3.3. Morphological Transitions of Microglia During Embryonic and Postnatal Development

Microglial progenitors have a typical ameboid morphology before entering the CNS, with a round-shaped soma and abundant cytoplasm with short, non-ramified processes. Such morphology is consistent with their primitive macrophage identity and migratory potential [25,62]. During this phase of migration, progenitors depend on integrin-mediated interactions with the extracellular matrix (ECM) that lines the pia surface. Talin-1-dependent integrin activation is particularly crucial as it enables firm adhesion and traction required for transmigration into the developing brain. Lack of talin-1 severely disrupts colonization of the CNS, highlighting its critical role in early microglial seeding. After entering the brain parenchyma, microglia progressively downregulate markers associated with the yolk sac, such as CD206 and F4/80. This marks the transition from a generic embryonic macrophage expression profile to a lineage-committed microglial fate [25,31].

Recently, single-cell RNA sequencing and immunofluorescence have identified two novel microglial subclusters in E14 mouse brains: EM1 (CD68-negative/Iba-1-positive) and EM2 (CD68- and Iba-1-double-positive). EM1 cells are relatively immature, highly proliferative, and without phagocytic markers, while the EM2 cells exhibit more branched morphologies, upregulate genes necessary for synaptic remodeling and neuronal differentiation, and are highly capable of synaptic phagocytosis, as evidenced by the colocalization with synaptophysin, along with increased expression of CD68 and complement-related markers [63].

As brain development continues, embryonic microglia progressively transition from an ameboid towards a ramified morphology, with increasing specialization within regions. Transcriptomic assessments conducted on human and murine developing brains reveal that early microglial populations preferentially express gene sets involved in regulating neuronal development, proliferation, and phagocytosis, such as scavenger and lipid-trafficking/handling receptors (e.g., *CD36*), which facilitate apoptotic and membrane clearance mechanisms, together with core regulators of synaptic pruning and neurodevelopmental remodeling, including complement pathway components (*C1QA*, *C1QB*, *C3*), fractalkine signaling elements (e.g., *CX_3_CR1*), and microglial activation receptors such as *TREM2*, all of which are highly associated with activity-dependent synapse refinement and neurogenic niche maintenance [9,63]. In contrast, at later developmental stages, microglial populations present with immune- and homeostasis-related gene signatures expressing *P2ry12*, *Tmem119*, *Cx3cr1*, *Il18*, *Fcrls*, *Siglech*, *Sall1*, and *Hexb*, often referred to as the microglial sensome [64]. These genes encode purinergic receptors, chemokine or cytokine receptors, including Cx_3_cr1 and Il18, immunoregulatory membrane proteins, and transcriptional regulators that are critical for the sensing, surveillance, and maintenance of microglial identity [3,65]. This molecular evolution aligns with increasing morphological and regional complexities, in which microglia adopt highly branched surveillant forms in gray matter areas such as cortex and hippocampus, while showing less ramified or elongated rod-like morphologies in white matter tracts like the corpus callosum and cerebellar peduncles, along with gene programs associated with lipid metabolism, axonal maintenance, and myelin remodeling. The white matter-associated microglia show elevated expression of markers such as Spp1 (osteopontin), ApoE, Gpnmb, and Lgals3, indicative of specialized roles in debris clearance, oligodendrocyte support, as well as myelin turnover that are critical during periods of active myelination and long-range circuit assembly [8,10,66].

The above transitions are not solely driven by intrinsic signals but are tightly regulated by environmental cues, including cytokines, growth factors, and ECM components, which help shaping microglial morphology and function. This developmental plasticity assures the proper integration of microglia into distinct regions of the CNS [37].

## 4. Functions of Developing Microglia Across Different Stages of Neurodevelopment

During early embryonic development, beginning at the fourth gestational week in humans and at E9.5 in mice, microglia promote neurogenesis by interacting with neural precursors, thus regulating their proliferation and survival through the secretion of IGF-1 and TGF-β, as well as through direct phagocytic uptake of apoptotic cells. This process is most profound at the peak of neurogenesis (first and second trimesters in humans; E10.5–16.5 in mice) [62,66]. When neurons start to migrate and axons extend (mid-gestation to early postnatal stage), microglia assist neurons in migration and axonal elongation by degrading the extracellular matrix and secreting cytokines and growth factors, such as TGF-β and IGF-1. Microglia also promote axonal pathfinding and the assembly of neural circuits through a bidirectional microglia-neuronal signaling using their TREM2 and CX_3_CR1 [62,67,68].

Microglia contribute substantially to the synaptogenesis throughout the late embryonic and early postnatal life, which is mainly mediated by the phagocytosis of supernumerary synapses. This process of synaptic pruning is modulated by complement proteins (C1q, C3), fractalkine signaling (CX_3_CR1), and TREM2 and reaches its peak during the early postnatal period (P5–15) in mice and late gestation to infancy in humans [27,67,68,69]. On the other hand, microglia were shown to directly contact developing dendrites, thus inducing dendritic spine filopodia [70,71]. Consistently, selective ablation of microglia during the development of the mouse motor cortex reduced both the formation and elimination of dendritic spines [72].

In white matter tracts, microglia regulate oligodendrogenesis and myelination of axons by engulfing the excess oligodendrocyte precursor cells (OPCs) and support the myelin formation, particularly before and during the onset of myelination (second postnatal week in rodents; early infancy in humans). This process is CX_3_CR1- and TREM2-dependent and maintains proper OPC/axon ratios for efficient myelination [64,68]. Microglia also influence astrogliogenesis by releasing cytokines and growth factors, mostly interleukin-6 () and leukemia inhibitory factor (LIF), which collectively facilitate the differentiation of astrocytes from neural stem/progenitor cells by activating the JAK/STAT and MAPK pathways [65]. Moreover, microglia regulate the number and distribution of astrocytes via C3/C3aR-mediated phagocytosis of excess cells. Disruptions of this pathway result in a high density of astrocytes and dysorganized glial grids [65]. During development, the stability of blood vessels is maintained by microglia, influencing angiogenic remodeling through the secretion of angiogenic factors, VEGF, and apoptotic endothelial cells clearance, especially during the angiogenic waves (mid-gestation to early postnatal stages) [44,73].

In addition, microglia promote maturation of neural circuits through the secretion of brain-derived neurotrophic factor (BDNF) in the process of experience-dependent synaptogenesis. Microglia-derived BDNF promotes the formation and stabilization of functional synapses by enhancing dendritic spine growth and strengthening synaptic transmission, thereby coupling neuronal activity to structural circuit refinement [72].

Because of the precisely timed alignment of microglial functions with the key milestones of neural development, these cells are highly sensitive to systemic and environmental influences, such as maternal immune activation, metabolic, and microbiome-derived signals. Previous studies have confirmed that disruption of these inputs during critical pre- or postnatal windows can alter microglial transcriptional maturation in a sex-dependent manner, bias circuit refinement, and render them uniquely vulnerable to neurodevelopmental disorders. Notably, microbiome-dependent regulation of microglial immune and metabolic programs persists beyond early life, underscoring a prolonged window during which microglial dysfunction can affect long-term brain function and vulnerability [74,75,76,77].

## 5. Microglial Contribution to the Pathology of Neurodevelopmental Disorders

The milestones of CNS development are closely entwined with the temporal and spatial processes of microglial proliferation, apoptosis, and maturation. Abnormalities, be they genetic, environmental, or inflammatory in nature, can cause long-term consequences for CNS structure and function, and it has become evident that immune activation plays a pivotal role in the neurodevelopmental trajectory. Microglial dysfunction, therefore, represents an important prognostic marker of disease development and progression, and the mechanisms behind microglial biology are increasingly emerging as possible therapeutic targets to mitigate or prevent neurodevelopmental diseases (Table 2) [78,79,80].

### 5.1. Microglial Impact on Epileptogenesis in the Immature Brain

Developmental epilepsies (or Developmental and Epileptic Encephalopathies (DEEs)) are a group of severe epilepsy syndromes that are characterized by early-onset, frequent, and often drug-resistant epileptiform activity. They are accompanied by substantial developmental impairment, which may present as developmental delay, regression, or a halt in developmental progress (plateauing). These developmental abnormalities likely result from an interplay between the physiological early network activity, the underlying (mainly genetic) pathology, and consequences of the seizures themselves (e.g., glutamate-induced toxicity), which could have a deteriorating effect on the neurodevelopmental processes. They are typically accompanied by various comorbidities, including intellectual disability, autism spectrum disorder (ASD), behavior abnormalities, movement disorders, and other extracerebral complications [81,82,83]. Conventional neurocentric approaches used in current antiepileptic treatment primarily help to control neuronal hyperexcitability or neurotransmitter release, without addressing the underlying glial role in epilepsy pathogenesis or the chronic neuroinflammation that can lead to seizures and impaired neurodevelopment. Thus, approximately one-third of patients are not well controlled with these approaches [83,84,85].

Given the crucial contribution of microglia in regulating synaptic and circuit remodeling as well as early brain maturation (see above), it is not surprising that their loss or dysfunction renders the developing brain vulnerable to excitation/inhibition imbalance [86], thereby pointing to a key role of microglia in the pathophysiology of epilepsy. Recently, disrupted microglial phenotype and function have been increasingly recognized as important factors in the pathogenesis of epilepsy (Table 2). The possible roles range from its onset to the progression, and influence neuroinflammation, synaptic structure, and early-life circuit development [85,87,88].

Among monogenic epilepsies, Dravet syndrome (DS) provides some of the most compelling evidence that microglial dysfunction could have an extremely early onset and a primarily developmental impact [89]. Due to loss-of-function SCN1A mutation and the resulting Nav1.1 channel deficits in the DS mouse model, functional impairments can be detectable at both early and later developmental stages. Chen et al. showed that primary microglia isolated from P1–P3 mutant pups display diminished phagocytic capacity and a shift toward a pro-inflammatory transcriptional state, indicating that microglial maturation and homeostatic function are altered. Upon activation (e.g., LPS stimulation), microglia exhibit moderately activated morphology and reduced proinflammatory cytokine expression, highlighting their immune dysfunction [89].

**Table 2:** Microglial contributions to neurodevelopmental disorder pathogenesis across defined developmental windows.

Targeted Disorder	Model/ Developmental Window	Microglial Phenotype and Dysfunction	Circuit-Level Mechanism	Pathophysiological Consequence	Key References
SCN1A-related DEE (Dravet syndrome)	knock-in mouse model (Scn1a^E1099X/+^)/P1–P3	Reduced phagocytic capacity; Reduced pro-inflammatory cytokine expression; Intermediate rather than fully activated morphology	Increased immature synaptic activity; Failure of E/I balance during hippocampal maturation	Early priming of epileptogenic vulnerability preceding overt seizures	[89]
SCN1A-related DEE (Dravet syndrome)	Scn1a^+/−^ mice/P15–P19	Increased microglial density and reactive morphology	Amplification of inflammatory signaling during interneuron failure	Exacerbated circuit instability and seizure burden	[90]
SCN2A-related DEE	human iPSC with Nav1.2-L1342P mutant channels/day in vitro (DIV) 0–36	Adaptive, homeostatic microglial responses	Suppression of neuronal hyperexcitability; Normalization of membrane properties	Protection against hyperexcitable network states	[91]
Tuberous Sclerosis Complex- associated epilepsy	Tsc1^GFAP^CKO, Tsc1^Cx3cr1-Cre^ CKO mice/ P14–P30 (pre-seizure)	Microglial mTOR hyperactivation; Metabolic and inflammatory dysregulation	Autonomous promotion of epileptogenic remodeling; Impaired synaptic refinement	Early circuit pathology and seizure susceptibility	[92,93]
SCN8A-related DEE	Scn8a^N1768D^ knock-in mice/P12–P20 (peri-seizure)	Minimal microglial activation; Astrocyte-dominant gliosis	Limited microglial contribution to the circuit remodeling	Highlights mutation-specific glial engagement	[94]
Perinatal hypoxia– ischemia-associated epilepsy	WT rats and mice/ P0–P7	Ameboid morphology; Elevated IL-1β signaling; excessive phagocytosis	Disrupted synaptic pruning; Impaired inhibitory circuit maturation	Increased epilepsy risk; long-term cognitive impairment	[95,96]
Maternal immune activation (MIA)	WT or CX_3_CR1^GFP/+^ transgenic mice (E12.5–E14.5)/ analyzed at E17–P7	Pro-inflammatory and complement-enriched transcriptome; Epigenetic priming	Impaired neurogenesis; Dendritic maturation defects; E/I imbalance	ASD; epilepsy susceptibility; cognitive deficits	[97,98]
Status epilepticus (SE)	WT and CX_3_CR1^GFP/+^ transgenic mice/ P7–P14	Exaggerated neuroinflammatory activation	Impaired synaptic maturation; persistent circuit instability	Neurodevelopmental comorbidities rather than seizure recurrence	[99,100]
Inflammation- associated ASD	WT (E12.5–E18) mice/ analyzed at P0–P7	Early pro-inflammatory activation followed by persistent transcriptional priming or hypo-responsiveness; complement enrichment; reduced process motility	Impaired neurogenesis and synaptic maturation; Disrupted activity- dependent pruning; Altered E/I balance	ASD core behaviors; increased seizure susceptibility; long-term cognitive impairment	[98,101,102]
MIA-associated ASD	WT or CX_3_CR1^GFP/+^ transgenic mice (E12.5–E18)/analyzed at E17–P7	Cytokine-driven microglial reprogramming (IL-6, IL-17A exposure); increased iNOS, IL-1β, Cxcl10, MHC I/II, C1q; epigenetic imprinting	Defective dendritic maturation and synaptic refinement during early postnatal development	Social communication deficits; repetitive behaviors; comorbid epilepsy risk	[97,102,103]
Perinatal inflammation-associated ASD risk	WT (E12.5–E18) mice/ analyzed at P0–P7	Sustained microglial inflammatory bias; altered cytokine signaling; impaired trophic support	Disrupted GABAergic maturation; Failure to stabilize developing cortical networks	ASD-like behaviors; cognitive and behavioral abnormalities	[95,101]
ASD-epilepsy overlap	WT mice/E17–P14	Biphasic microglial response: early hyperactivation followed by reduced synaptic surveillance capacity	Persistent E/I imbalance due to defective pruning and synapse stabilization	Co-occurring ASD and epilepsy phenotypes	[98,102,104]

As animals reach the developmental period in which spontaneous seizures emerge (≥P20), these early microglial abnormalities coincide with marked synaptic immaturity in the dentate gyrus, including excessive immature excitatory activity and disrupted synaptic organization [105]. Although direct causality has not been formally established, the temporal convergence of reduced microglial phagocytosis, altered inflammatory signaling, and impaired synaptic refinement strongly suggests that microglial dysfunction contributes to impaired excitation/inhibition (E/I) balance during hippocampal circuit maturation. In concert with this mechanistic perspective, a broader body of evidence indicates that compromised microglial activation can lead to neuronal injury, synaptic disruption, and network hyperexcitability in experimental epilepsy models, independent of primary neuronal pathology [106].

Microglial phagocytosis involves a complex interplay of “find me” and “eat me” signals mediated, among other intracellular signals, by phagocytic receptors (such as complement C1q/C3 and MerTK). In epilepsy, all of these processes are disrupted; alterations in complement signaling and decreased expression of phagocytosis-related proteins compromise microglia’s efforts to remove redundant or dysfunctional synapses [107]. Furthermore, in DS, the impaired GABAergic signaling not only reduces inhibition in the neural network but also disrupts microglial maturation and function, affecting their “eat me” responsiveness as well as synaptic pruning efficiency [88]. This, in turn, creates a vicious cycle where, upon failure in microglial pruning, the number of immature synapses increases, promoting hyperexcitability of neurons and seizure events. Simultaneously, persistent seizures and neuroinflammation further impact the stability of microglial functions [88,89]. Taken together, cross-model evidence establishes DS as a condition in which microglia function not merely as secondary responders to seizures but as active contributors to the miswiring of early (hippocampal) neural networks [89,90,105].

By contrast, an opposite dynamic is at play in SCN2A developmental epilepsy, where microglia seem to exert a protective, homeostatic role. Within the human-induced pluripotent stem cell-derived neuron-microglia co-culture with an SCN2A L1342P mutation, microglia have been shown to sense and then adapt to changes associated with neuronal hyperexcitability [91]. Que et al. demonstrated that microglia cocultured with hyperexcitable mutant neurons undergo morphological alterations (increased branch length) and enhanced process-specific calcium signaling. Rather than enhancing inflammation, their presence reduces ongoing neuronal activity and sodium current density, thus reducing hyperactivity. A possible molecular mechanism for such dichotomic function of microglia was proposed by Badimon et al. [86], who showed that microglial processes are recruited to (hyper)active tripartite synapses via neuronal/astrocytic release of ATP. This ATP is then catabolized by the microglial ATP/ADP hydrolyzing ectoenzyme CD39 into AMP, which, in turn, is converted to adenosine by ecto-5′-nucleotidase CD73, expressed on the microglial surface. In this way, the proximity of a microglial process to a (hyper)active glutamatergic synapse directly translates into the amount of adenosine available to bind to the postsynaptic A1 receptors, decreasing neuronal excitability. Thus, microglial hyperramification in synapse proximity increases A1-mediated inhibition, whereas microglial hypertrophy and process withdrawal in the course of activation decrease A1-mediated inhibition. Via such bidirectional communication, microglia can detect the excess neuronal activity and respond by affecting their functional state, exerting a direct, homeostatic influence on developing neuronal circuits [91]. Interestingly, OPCs seem to play a similar role [108], thus pointing to an emerging pan-glial phenomenon.

Consistent with the above mechanistic explanation, studies on early epileptogenesis in Tuberous Sclerosis Complex (TSC) disorder suggest another paradigm in which microglia become primary drivers of early circuit pathology. TSC results from loss-of-function mutations in *TSC1* or *TSC2*, leading to constitutive hyperactivation of mTOR, an intracellular signaling pathway that regulates microglial metabolism, phagocytosis, and cytokine production. In juvenile *Tsc1* conditional mouse models, robust microgliosis, enlarged microglial somata, and hypertrophy, along with transcriptional signatures indicative of heightened inflammatory and metabolic activation across hippocampal and cortical regions, are detectable well before the full manifestation of seizures [93]. Notably, microglia-specific deletion of *Tsc1* shows that mTOR hyperactivation within microglia provokes morphological abnormalities and facilitates epileptogenic remodeling, even in the absence of neuronal *Tsc1* loss. This positions microglia-intrinsic mTOR dysregulation as a mechanistic link between genetic mutation and disrupted circuit maturation in early-life seizure susceptibility [92].

Interestingly, studies in the SCN8A developmental epileptic encephalopathy clarified that microglial involvement across all genetic epilepsies is not uniform. Although SCN8A mutations cause severe early-onset epilepsy and pronounced network hyperexcitability, developmental analysis revealed minimal morphological or transcriptional changes in microglia around seizure onset. Instead, astrocyte reactivity dominated the glial landscape. This negative finding is important, highlighting the possible mutation-specific engagement of microglia [94].

Perinatal inflammation represents a prominent early-life risk factor for childhood epilepsy, where microglia likely play a key role. Perinatal injury, which may result from either hypoxia and/or maternal immune activation, prompts microglia to shift toward a pro-inflammatory phenotype, consequently manifesting in both acute and chronic alterations in their gene expression, morphology, and cytokine signaling. This shift is accompanied by an increase in seizure susceptibility and compromised long-term neurodevelopment [95,101]. Hypoxic-ischemic injury at childbirth rapidly activates microglia. This can be confirmed through morphological shifts toward an ameboid phenotype, upregulated expression of the pro-inflammatory cytokines IL-1β and TNFα, and enhanced phagocytic activity. This acute inflammatory response impairs normal synaptic pruning and GABAergic signaling, initiating a cascade of long-term changes in neural circuit maturation and promoting network excitability. This sets the stage for seizures and cognitive abnormalities. Early interference inhibiting the IL-1β signaling cascade can prevent such long-term disruptions, clarifying how microglia-driven inflammation might link perinatal asphyxia to neurodevelopmental abnormalities [95,96].

Maternal immune activation (MIA), commonly modeled using viral/bacterial mimetics such as poly(I:C) or LPS at mid-gestation (typically E12.5–E14.5), allows maternal cytokines such as IL-6 and IL-17A to cross the placenta and directly affect the developmental trajectory of fetal microglia [98,101,102]. Within 24–48 h of maternal cytokine exposure, fetal microglia undergo a transition to a pro-inflammatory and complement-enriched transcriptome. This involves increased expression of genes encoding iNOS, IL-1β, Cxcl10, and MHC class I/II molecules, as well as C1q, with reduced process motility and impaired neurogenic support [97,98,102,109]. Epigenetic reprogramming of microglia during this time window further amplifies their pro-inflammatory responsiveness to subsequent insults [103].

Interestingly, this acute phase of hyperactivation is followed by a secondary phase of blunted microglial reactivity or hypo-responsiveness, characterized by suppressed inflammatory responsiveness to stimuli and reduced capacity for synaptic refinement, which emerge in the late fetal period and persist into the early postnatal window. This biphasic pattern can impair neurogenesis, dendritic maturation, and E/I balance [102,104,109], thus enhancing susceptibility to early-life epilepsy, ASD, and long-term cognitive abnormalities [98,102,104].

The heightened vulnerability of developing microglia to inflammatory stimuli is especially evident following status epilepticus (SE), which induces rapid and robust microglial activation in the immature brain. This activation is manifested by increased microglial cell numbers, morphological alterations consistent with a reactive phenotype, and overproduction of pro-inflammatory cytokines, including TNFα and IL-1β [110,111]. Notably, the timing of developmental SE critically shapes this response. In mice, experimental SE induced during the first postnatal week is associated with relatively minimal microglial activation and limited neuronal injury, whereas seizures during the second postnatal week (P10–P14, coinciding with the peak in postnatal microglial density) strongly provoke neuroinflammatory responses, impair synaptic maturation, and enhance long-term circuit instability [99,100]. Broad pharmacological suppression or extensive depletion of microglia after SE can ameliorate subsequent neurobehavioral deficits without consistently preventing recurrent spontaneous seizures [112,113].

**Figure 1:**
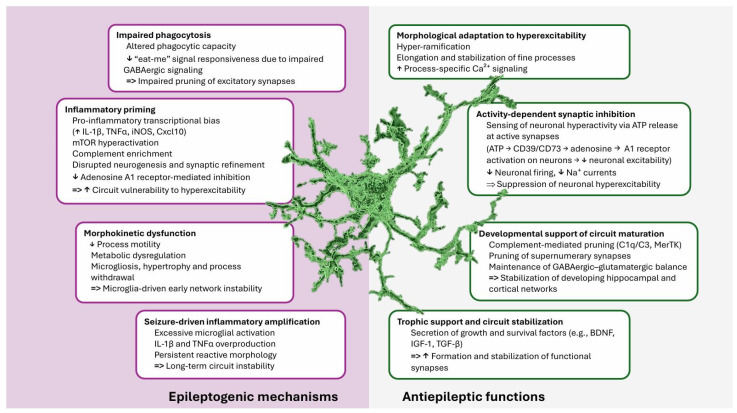
Bidirectional microglial contribution to neonatal/juvenile epileptogenesis. Central microglial rendering was generated using Imaris 10.2 (Andor Technology Ltd., Belfast, Great Britain, UK) for illustrative purposes. For key references, see Table 2.

Overall, these studies identify microglia as developmentally sensitive regulators of epileptogenesis whose roles vary depending on the genetic context, developmental timing, and inflammatory status. Rather than acting uniformly as secondary responders to seizures, microglia can either cause circuit miswiring or exert homeostatic control over neuronal excitability, converging on shared mechanisms of synaptic refinement and E/I balance. This highlights the need for stage- and mutation-specific therapeutic strategies that restore microglial function rather than globally suppressing neuroinflammation (Figure 1).

### 5.2. Microglia-Mediated Developmental Pathology in Autism Spectrum Disorder

Findings from human studies increasingly support a pivotal role of microglial dysregulation in ASD, confirming that the immune and synaptic impairments observed in experimental models are clinically relevant. Indeed, postmortem analyses of ASD brains consistently show altered microglial density, morphology, and transcriptional profile, mostly characterized by persistent inflammatory activation and impaired interactions with neuronal and synaptic compartments [78,114]. Evidence for increased microglial activation was also provided by in vivo PET imaging in young human adults with ASD, which shows that microglial alterations are not transient developmental phenomena but may persist across the lifespan [115]. Thereby, these human data align with the notion that microglial dysfunction is an enduring feature of ASD pathology, rather than a secondary consequence of behavioral impairment.

Complementing these observations, systems based on human-induced pluripotent stem cells (iPSCs) have yielded critical insight into underlying mechanisms. iPSC-based neuron-microglia co-culture models demonstrate suppressed microglial sensing of neuronal activity, dysregulated inflammatory responses, and aberrant synaptic remodeling in ASD-associated genetic backgrounds [116]. These human-relevant platforms bridge the gap between postmortem pathology and animal models, demonstrating that during early circuit formation, microglial dysfunction can emerge in either a cell-autonomous or cell-interactive manner. Of course, animal studies remain essential to resolve developmental timing and mechanistic specificity (Table 2). Paralleling developmental epilepsies, ASD-relevant rodent analyses show that perturbation of microglial programming during embryonic and perinatal development, most notably following maternal immune activation, induces persistent microglial dysfunction that precedes aberrant synaptic wiring and later behavioral phenotypes [97,102,104]. One possible molecular pathway likely acts via systemic inflammation, as increased levels of pro-inflammatory cytokines, including TNF-α, IL-1β, IL-6, IL-8, and interferon-gamma (IFN-γ) have been consistently observed in the peripheral blood of ASD patients [117,118]. In the in vivo mouse brain, increased systemic levels of pro-inflammatory cytokines cause both activation of microglia and hyperactivity of cortical neural networks [119,120].

Furthermore, recent studies indicate that microglial cell-intrinsic regulatory mechanisms are sufficient to produce ASD-relevant phenotypes. Epigenetic disruption of microglial homeostasis by deficiency in ARID1A (encodes AT-rich interactive domain-containing protein 1A, which regulates chromatin structure and gene transcription in an ATP-dependent manner) causes impaired microglial-neural progenitor interactions and ASD-relevant phenotypes in mice. This indicates that microglial dysfunction by itself can disrupt neurodevelopmental trajectories [102]. This notion is supported by experiments in mice, selectively lacking the key autophagy-related gene *atg7* in myeloid cells [121]. In atg7-deficient microglia, impaired autophagy was paralleled by reduced phagocytosis, altered anatomical and functional brain connectivity, and autistic behaviors. In addition, genetic variants in human microglia-specific genes such as *CX_3_CR1* and *TREM2* were also associated with an increased risk of ASD [117,122]. Consistently, preclinical studies in Cx3cr1- as well as Trem2-deficient mice also found ASD-like symptoms such as deficits in social interactions, increased repetitive behavior, poor functional brain connectivity, and impaired synaptic transmission (Figures 1, 3 and 4 in refs. [118,123]).

On a synaptic level, ASD-like traits are associated with aberrant synaptic pruning, whereby both impaired and excessive synaptic pruning are observed [124]. In Trem2-deficient mice, for example, impaired synapse elimination is paralleled by a reduction in the number of hippocampal microglia, excess of glutamatergic synapses and neural network hyperactivity [123]. Similar observations were made in the cortex of atg7-deficient mice [121]. In SCN2A-deficient mice, microglia-mediated synaptic overpruning was accompanied by reduced hippocampal synaptic spine density as well as impaired learning, memory, and excitatory synaptic transmission [125]. Of note, in these mice synaptic transmission and spine density were partially restored by microglia ablation using orally administered CSF1R inhibitor PLX3397. The authors further verified their findings in human cerebral organoids, expressing an ASD-associated SCN2A protein-truncating mutation to observe a similar overpruning of excitatory synapses [125].

In conclusion, the described findings are consistent with a growing body of evidence placing ASD and epilepsy on a continuum of neurodevelopmental disorders. Many ASD-associated genetic mutations, including SCN2A, confer high risk for early-life epilepsy, and both conditions are characterized by neural network hyperactivity, disruptions in E/I balance, synaptic maturation, and circuit stability. Microglia sit at the nexus of such pathophysiological mechanisms. Aberrant microglial pruning, systemic inflammation, or impaired homeostatic signaling during early development can bias neural circuits toward hyperexcitability, increasing vulnerability to both epileptogenesis and autistic phenotypes.

Within this epilepsy-autism overlap framework, microglia emerge not merely as modulators of inflammation but as active determinants of developmental circuit fate. The timing, direction, and genetic context of microglial dysfunction appear to dictate whether outcomes manifest predominantly as ASD, epilepsy, or their frequent comorbidity. This shared mechanistic substrate underscores the importance of microglia as a unifying cellular target for understanding and potential intervention in neurodevelopmental disorders spanning the autism-epilepsy spectrum.

## Data Availability

No new data were created or analyzed in this study. Data sharing is not applicable to this article.
